# A Multicenter Preclinical MRI Study: Definition of Rat Brain Relaxometry Reference Maps

**DOI:** 10.3389/fninf.2020.00022

**Published:** 2020-05-19

**Authors:** Tristan Deruelle, Frank Kober, Adriana Perles-Barbacaru, Thierry Delzescaux, Vincent Noblet, Emmanuel L. Barbier, Michel Dojat

**Affiliations:** ^1^INSERM, U1216, Grenoble Institut des Neurosciences, Université Grenoble Alpes, Grenoble, France; ^2^CNRS, CRMBM, Aix-Marseille Université, Marseille, France; ^3^UMR-9199, CNRS, CEA-MIRCen, Paris Saclay, Fontenay-aux-Roses, France; ^4^CNRS, ICube – IMAGeS, Strasbourg University, Strasbourg, France

**Keywords:** MRI, brain imaging, rat, quantitative imaging, digital atlas

## Abstract

Similarly to human population imaging, there are several well-founded motivations for animal population imaging, the most notable being the improvement of the validity of statistical results by pooling a sufficient number of animal data provided by different imaging centers. In this paper, we demonstrate the feasibility of such a multicenter animal study, sharing raw data from forty rats and processing pipelines between four imaging centers. As specific use case, we focused on T1 and T2 mapping of the healthy rat brain at 7T. We quantitatively report about the variability observed across two MR data providers and evaluate the influence of image processing steps on the final maps, using three fitting algorithms from three centers. Finally, to derive relaxation times from different brain areas, two multi-atlas segmentation pipelines from different centers were performed on two different platforms. Differences between the two data providers were 2.21% for T1 and 9.52% for T2. Differences between processing pipelines were 1.04% for T1 and 3.33% for T2. These maps, obtained in healthy conditions, may be used in the future as reference when exploring alterations in animal models of pathology.

## Introduction

In the clinical domain, multicenter studies are common. Their main objective is to gather data from a sufficient number of patients in a reasonable period of time to improve the statistical power and consequently the robustness of the reported results. Multicenter studies also set the basis for developing and validating quantitative and reproducible imaging biomarkers. Similarly, there are several well-founded motivations for animal population imaging: optimization of costs, reduction of experimentation duration, and improvement of quality of science, notably by the use of sufficiently large animal cohorts for ensuring the validity of statistical results [see the special Lab Animal focus on reproducibility in animal research (Prescott and Lidster, 2017)]. This domain is still in its infancy, and we may expect it to develop in the near future. Consequently, only few tools are available to facilitate preclinical data pooling. Moreover, there is a clear lack of large actions for standardization of image acquisition conditions and post-processing techniques. Finally, there are no reliable commonly adopted preclinical imaging biomarkers for differentiating normal vs. pathological conditions. The aim of the present work was to assess the feasibility of multicenter preclinical studies in order to define robust biomarkers. We thus considered a specific use case: quantitative T1 and T2 mapping of the healthy rat brain at 7T.

The T1 and T2 relaxation times are tissue- and region-dependent. As they may reflect micro-anatomical alterations, they are biomarkers for various pathologies (Deoni, 2010). To map these relaxation times, series of weighted images are acquired with varying acquisition parameters such as echo time (TE), inversion time (TI) or flip angle. Voxel-per-voxel fitting of a model equation (with generally two or three parameters) to these series is then used to calculate relaxation time maps (Guilfoyle et al., 2003; de Graaf et al., 2006; Wright et al., 2008). Alternatively, newer fingerprinting methods based on the use of dictionaries currently emerge (Gao et al., 2015). A large number of monocentric studies measured T1 and T2 in rodents, at different magnetic fields and using different acquisition protocols. They show that T1 relaxation time increases with magnetic field while T2 relaxation time decreases (de Graaf et al., 2006; van de Ven et al., 2007; Wright et al., 2008). Some studies also reported regional values (Cremillieux et al., 1998; Barbier et al., 2005; Del Bigio et al., 2011; Gigliucci et al., 2014; Suleymanova et al., 2014; Koundal et al., 2015; Liachenko and Ramu, 2017; Behroozi et al., 2018), but no consensus has been reached yet about values of reference for specific rat brain regions. To define such reference maps, a large number of brain structures or regions should be considered and a sufficient number of animals should be included to reflect inter-individual variability. In this context, a multicenter study is relevant.

For Human studies, to facilitate data storage, data sharing and data processing with specific pipelines, several infrastructures have been proposed such as COINS (Landis et al., 2016), LORIS + BRAIN (Das et al., 2016) or LONI (Rex et al., 2003) for Neuroimaging multicenter studies [see recent works in this field (Dojat et al., 2017)]. These infrastructures support the “Open Science” approach, an international action to improve the use of resources, to ease study replication, and to strengthen the validity of scientific results (NAP, 2018). This promotes studies on very large cohorts (e.g., Adhikari et al., 2019), the development of reference databases [see for Alzheimer disease (Li et al., 2017), Parkinson disease (Chahine et al., 2018) or Human connectome project (Hodge et al., 2016)] and fair and robust comparison of image processing solutions (Commowick et al., 2018). Here, we propose to use an extension of the SHAring NeurOImaging Resources environment (Barillot et al., 2016) for storing preclinical imaging data (Small Animal Shanoir, SAS)^1^ in conjunction with the VIP^2^ architecture, a platform dedicated to the execution of image processing pipelines (Glatard et al., 2013). We quantitatively report about the variability observed across two data provider centers and evaluate different image processing pipelines. We finally discuss the feasibility of small animal population studies. To promote data sharing in the preclinical domain, raw and processed datasets as well as processing pipelines have been made available (see section “Discussion”).

## Materials and Methods

### Distribution of Tasks Between Centers

Two centers, GIN (C1) and CRMBM (C2) hosted the animals and performed brain MRI acquisitions. Three centers C2, MIRCen (C3) and ICube (C4) provided processing pipelines.

### Animals

Twenty Sprague Dawley rats (male, Janvier Labs, Paris France, mean weight 279 ± 40 g [(min: 249.5 g, max: 314 g), details in Supplementary Table S1] were scanned in two imaging centers (C1 and C2). Animals were anesthetized with isoflurane [2% in air at C2 and 2% in a mixture of Air and O_2_ (7:3) at C1] that was delivered via a nose cone during the experiment. Animals were positioned in prone position on an animal bed (Bruker Biospin, Ettlingen, Germany). Breath rate was monitored using a pneumatic pillow sensor placed under the abdomen. Body temperature was measured with a rectal probe and maintained in the normal range at 36.2 ± 1.4°C using a heated blanket. To control acquisition reproducibility, three rats were scanned twice, one rat (Subject 32, S32) at C2 at a 2-day interval and two rats (Subject 21, S21 and Subject 22, S22) at C1 at a 3-day interval. All experiments were approved by the local ethics committee of each center and were in full compliance with the guidelines of the European Union (EUVD 86/609/EEC) for the care and use of the laboratory animals. Experiments were performed under permits from the French Ministry of Agriculture (n° 380945 and A3851610008 for experimental and animal care facilities for C1 and G130555 for C2).

### MRI Protocol

Acquisitions were performed on 7T horizontal Bruker scanners using the same MR sequences and parameters at data provider centers C1 and C2 (aC1 and aC2, respectively, see details in Table 1). Preliminary *in vitro* experiments were performed at C1 and C2 in order to select the best sequences to use, with the objective to minimize acquisition time and geometric artifacts, and to maximize spatial resolution. A 3D MDEFT sequence (with Inversion Preparation as MPRAGE) was chosen for T1 mapping (REF)^3^. Multi-Slice Multi-Echo (MSME) was chosen for T2 mapping. For T1 mapping, the MPRAGE sequence was run seven times with incremental inversion times (TI) and for T2 mapping, a 3D MSME sequence with 28 echo times (TE) was used (DiFrancesco et al., 2008; Liu et al., 2011). Main sequence parameters are shown in Table 1. Total experiment duration per animal was about 2 h.

**TABLE 1:** Equipment characteristics, Fitting and Segmentation methods. In the model equations, A, B, T1 and T2 are the parameters to be estimated.

*Acquisition*	GIN (aC1)	CRMBM (aC2)
Scanner model	Bruker BioSpin MRI GmbH – Biospec 70/20 7.0T	Bruker BioSpin MRI GmbH – Pharmascan B-C 70/16US 7.0T
Transmitting coil	Linear Volumetric Coil 72 mm	Quadrature Resonator 72 mm
Receiving coil	Rat Head Coil (Surface) 1 channel	GIN coil
Gradient system	BGA12S2 (110 mm, max 660 mT/m, slew rate 4570 T/m/s)	BGA 9SHP (90 mm, max 750 mT/m, slew rate 6840 T/m/s)
T1 mapping	MPRAGE sequence; TI = 247, 408, 674, 1112, 1838, 3030, and 5000 ms; TR = 6500 ms	
T2 mapping	3D MSME; TE = (8:224 ms); TR = 600 ms	
	FOV: 2.7 cm × 2.7 cm × 2.8 cm, matrix size: 128 × 128 × 66, spatial resolution: 211 μm × 211 μm × 424 μm	

*Fitting*	GIN (fC1)	CRMBM (fC2)	MIRCen (fC3)
Software	MATLAB R2015a	ImageJ v1.51	BrainVISA/Python
Equation T1	$y=|A.\left(1-2B.\exp\left(-\frac{TI}{T1}\right)\right)|$	$y=|A.\left(1-2B.\exp\left(-\frac{TI}{T1}\right)\right)|$	$y=|A.\left(1-2B.\exp\left(-\frac{TI}{T1}\right)\right)|$
Equation T2	$y=|A.\exp\left(-\frac{TE}{T2}\right)|$	$y=|A.\exp\left(-\frac{TE}{T2}\right)|$	$y=|A.\exp\left(-\frac{TE}{T2}\right)|$

*Segmentation*	ICube (sC4)	MIRCEN (sC3)
Brain masking	Matlab/spm12	
Bias field correction	N4	
Registration	Non-rigid [ANTS (Avants et al., 2010)]	Rigid + Block-Matching affine (Lebenberg et al., 2010)
Number of atlases	11	12
Probability rule	Majority voting	

*See text for details.*

### Data Processing and Analysis

**FIGURE 1:**
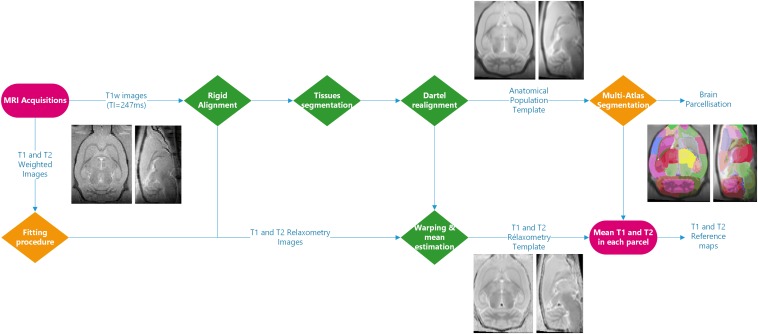
Processing workflow: the processing steps performed using SPM12 are shown in green. Three different fitting procedures and two implementations of the multi-atlas segmentation method were introduced. The inserted MR images are representative of the imaging processing outputs.

Figure 1 illustrates the complete image processing workflow. Several preprocessing steps were performed using SPM12^4^ and MATLAB R2015a. Briefly, Bruker files were first converted into NIFTI images using home-made software. All anatomical images were rigidly realigned on a study-specific rat template. Tissue segmentation was performed for each animal (Ashburner and Friston, 2005) based on our study-specific, tissue prior, template. Using the Dartel registration algorithm (Ashburner, 2007) adapted to rat images, these tissue images were non-rigidly registered.

The individual deformation field was then applied to the corresponding individual anatomical and relaxometry images, and all these images were averaged to compute anatomical and relaxometry mean templates. Additionally, T1 and T2 weighted raw images were separately processed using three different fitting pipelines. The differences between these pipelines developed at C1, C2, and C3 (fC1, fC2 and fC3, respectively), are summarized in Table 1. All algorithms performed non-linear pixel-per-pixel fitting for each voxel independently. Negative values and values greater than 3000 ms for T1 and 300 ms for T2 were discarded. The optimization algorithm was Levenberg-Marquardt for fC1 and fC3 and the Simplex algorithm provided with ImageJ for fC2 (Nelder and Mead, 1965). The three T2 estimation procedures rely on the same model equations.

**FIGURE 2:**
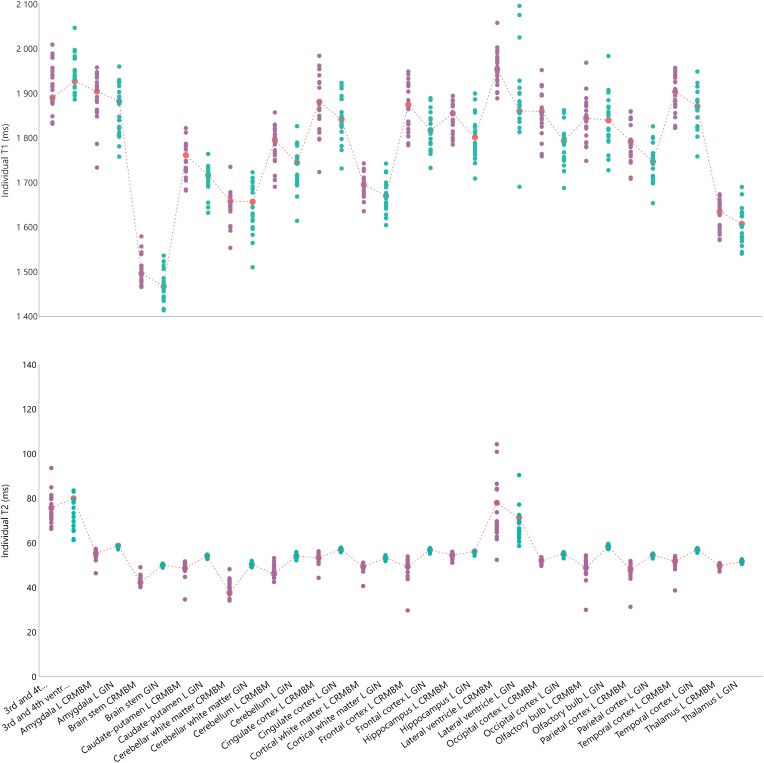
Individual relaxation times for 16 regions of interest for the left hemisphere including 3 regions overlappping the two hemispheres. **Top:** Individual T1 values. **Bottom:** Individual T2 values. Green circles for aC1 values; purple circles for aC2 values and corresponding mean values indicated with a red mark and dash-line. The fC1 fitting pipeline and the sC4 multi-atlas segmentation were used.

Rat brain parcelation was performed using two multi-atlas approaches similar to the one proposed by Lancelot et al. (2014), for a precise and reproducible delineation of brain structures in preclinical *in vivo* imaging. A maximum probability automatic delineation was obtained by the fusion of several manually delineated images placed in a common space and constituting the multi-atlas dataset. This dataset was registered to the native space of the MR image to segment. At each voxel, the most likely label in the dataset was selected by a maximum probability rule. Two versions of this approach were implemented and executed, one at C3 (sC3) using BrainVISA^5^ and one at C4 (sC4) using VIP (Glatard et al., 2013), which differ in some aspects as detailed in Table 1. Twenty-nine brain regions were defined (see Figure 2). MR data initially stored in the SAS database were automatically sent to the VIP processing platform and processed results were seamlessly stored [see more details and the Figure 8 in Commowick et al. (2018) for database and computing platform integration with Shanoir].

The twelve pipelines combining data acquisition (aC1 and aC2), fitting (fC1, fC2, and fC3) and segmentation (sC3 and sC4) were compared. The processing pipelines and data are available on request (see section “Discussion”). Statistical analysis was performed with MS Excel 2010 and Real Statistics^6^. Because most of the samples did not present a normal distribution (Shapiro–Wilk test), non-parametric tests were performed.

## Results

### Inter-Subject Data Variability

For each individually segmented rat brain, we computed the mean T1 and T2 values for the 29 regions (13 in each hemisphere and 3 non-lateralized regions). Figure 2 shows these values for each region of the left hemisphere and for each rat, computed using the fC1 fitting pipeline and the sC4 multi-atlas segmentation.

We note that for both T1 and T2 values, the largest dispersion is for the ventricles (lateral, 3rd and 4th ventricles). On average, the differences between the minimum and maximum values of each region are 170 ms for T1 and 11 ms for T2 (left hemisphere regions, excluding ventricles). We obtained similar results for the right hemisphere (169 and 9.3 ms, respectively) and with the other pipelines (e.g., Supplementary Figure S1 for an example using the fC2 fitting pipeline and the sC3 multi-atlas).

### Inter-Center Acquisition Reproducibility

We studied the differences between T1 and T2 values computed from data acquired at aC1 and aC2 using the same pipelines. Between the two centers, the differences (see Supplementary Figure S2) were significant for T2 (Mann–Whitney test *p* < 2 10^–4^, with 9% mean error), but not for T1 (Mann–Whitney test *p* = 0.02, with 2% mean error).

### Control of Intra-Center Acquisition Reproducibility

We studied the scan-rescan differences for each subject (see Supplementary Figure S3). The differences were less than two standard deviations in all ROIs of all subjects, except in the left lateral ventricle (S32), the right olfactory bulb (S21) and the 3rd and 4th ventricles (S22). For T2, large differences were found in the left lateral ventricle (S32), and in the 3rd and 4th ventricles (S21 and S22). A Wilcoxon statistical test was run to compare the results between the first and second MR acquisition for T1 and T2, for each rat, and for different pipeline combinations. Differences were not statistically significant. The parameters of the linear regression between the results from the scan-rescan experiment for each subject were close to the identity curve with a R2 coefficient for T1 equal to (0.89, 0.89, and 0,94) and for T2 (0.88, 0.97, and 0.99) for rats S32, S32, and S22, respectively.

### Fitting Pipeline Comparison

**FIGURE 3:**
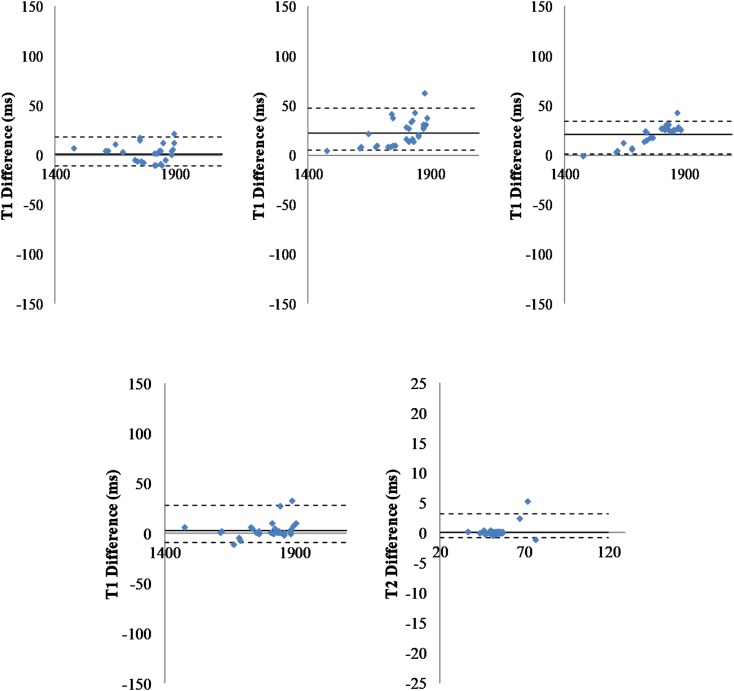
Comparison of pipelines. **Top:** Fitting pipelines. Bland-Altman representation of difference between T1 relaxation times for all regions of interest (*n* = 29), averaged across all animals (*n* = 40) and obtained with three different fitting pipelines. Left: T1 differences for fC1 minus fC2; Middle: T1 differences for fC1 minus fC3; Right: T1 differences for fC2 minus fC3. The sC4 was used for segmentation. Solid line: Mean difference. Dash-lines: ± two standard deviations. **Bottom:** Segmentation pipelines: Bland-Altman graph of the T1 (left) and T2 (right) differences measured for all regions of interest (*n* = 29), averaged all animals (*n* = 40) and obtained with two different segmentation pipelines (one point per region of interest). The fC1 fitting pipeline was used. Solid line: mean difference. Dash-lines: ± two standard deviations.

To compare results obtained with the three fitting pipelines, regressions were computed and indicated good consistency (see Figure 3, Top). The linear regression parameters were *y* = 0.99x + 5.11 (*R*^2^ = 0.99) for T1 (C2f) vs. T1 (C1f), *y* = 0.92x + 111.9 (*R*^2^ = 0.98) for T1 (C3f) vs. T1 (C1f), and *y* = 0.93x + 101.94 (*R*^2^ = 0.99) for T1 (C3f) vs. T1 (C2f).

Similar results were obtained for T2 (see Supplementary Figure S4) and using two different segmentation pipelines (see Supplementary Figure S4). A Wilcoxon statistical test was run for each pair of pipelines. Differences were not statistically significant (mean error to identity for T2: 3.33%, for T1 1.04%).

### Segmentation Pipeline Comparison

To compare results obtained using the two segmentation pipelines, regressions were computed. As for the fitting pipelines, Figure 3 (bottom) shows a good concordance between the methods. The linear regression parameters were *y* = 1.012x-16.92 (*R*^2^ = 0.99) for T1 (C3s) vs. T1 (C4s) and *y* = 1.0583x-2.9489 (*R*^2^ = 0.99) for T2 (C3s) vs. T2 (C4s).

*P-*values obtained with a Wilcoxon test were 0.823 and 0.994 for T1 and T2, respectively. These values were obtained via the two implementations of the same multi-atlas segmentation method (mean error to identity of 0.24% for T1 and 0.81% for T2). Similar results were obtained when using the two other fitting methods (e.g., Supplementary Figure S4 for fC2).

### Comparison With Literature

**FIGURE 4:**
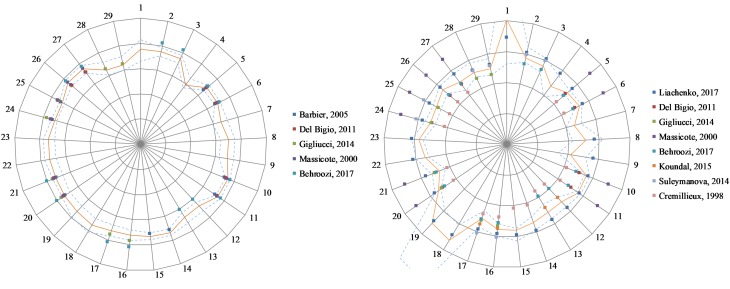
Relaxation values for this study and five published studies (see References list for details) in 29 regions of interest. **Left:** T1 relaxation values. Scale: 500 ms/division. **Right:** T2 relaxation. Scale: 15 ms/division. The orange line indicates results from this study. Dash lines indicate ± one standard deviation from our results. 1: 3rd and 4th ventricles, 2: Amygdala L, 3: Amygdala R, 4: Brain stem, 5: Caudate-putamen L, 6: Caudate-putamen R, 7: Cerebellar white matter, 8: Cerebellum L, 9: Cerebellum R, 10: Cingulate cortex L, 11: Cingulate cortex R, 12: Cortical white matter L, 13: Cortical white matter R, 14: Frontal cortex L, 15: Frontal cortex R, 16: Hippocampus L, 17: Hippocampus R, 18: Lateral ventricle L, 19: Lateral ventricle R, 20: Occipital cortex L, 21: Occipital cortex R, 22: Olfactory bulb L, 23: Olfactory bulb R, 24: Parietal cortex L, 25: Parietal cortex R, 26: Temporal cortex L, 27: Temporal cortex R, 28: Thalamus L, 29: Thalamus R.

Figure 4 shows the T1 and T2 values obtained in this study and those reported in literature. When literature only reported one cortical ROI, that value was replicated for all cortical ROIs of this study.

## Discussion

In this study, we report a multicenter T1 and T2 mapping of the rat brain at 7 Tesla. Four centers were involved: two contributed to data acquisition, and two centers provided two multi-atlas segmentation pipelines to derive relaxation times per brain region. To facilitate the pooling of the preclinical MR datasets, we used the SHAring NeurOImaging Resources (Shanoir) environment, initially developed for the web-oriented management of collaborative neuroimaging projects in Humans, and recently extended for preclinical studies (Small Animal Shanoir, SAS).

### Data Pooling

We successfully combined MR data acquired on forty rats in two different centers. We took advantage of the fact that the fleet of MR scanners for preclinical studies tends to be more uniform across labs than Human imaging systems. Indeed, the MR scanners in both centers were from the same manufacturer (Bruker Biospin) and, as indicated in Table 1, there were very few differences between the two systems. The acquisition sequence parameters could thus be set identical in both centers. Consequently, Figure 3 shows a small dispersion of individual T1 and T2 values in brain regions, except for the ventricles (lateral and 3rd and 4th ventricles). Moreover, we noted a good intra-center reproducibility for T1 and T2 values obtained on three rats. There was no significant difference in the scan-rescan experiment of each rat. Good reproducibility was also obtained when using different processing solutions (Supplementary Figure S3). The observed differences for ventricles could be a consequence of the small number of voxels of these structures compared to other regions (3500 and around 11000 voxels for lateral and 3rd and 4th ventricles, respectively, versus 33000 voxels on average for the other structures) and to the large differences in relaxation times between brain tissue and cerebrospinal fluid. Moreover, in T1-weighted images, the contrast between ventricles and tissue was low. This made the registration process more prone to errors. Also, small movements of the cerebrospinal fluid in the ventricles during acquisition may lead to a biased estimation of relaxation times in these regions, especially for T2, which exhibits the largest difference between tissue and ventricles. Moreover, for some rats (*n* = 3), ventricles were found dilated. Such dilation may impact the global results. T1 values computed from data acquired at C1 or C2 using different fitting and segmentation pipelines were not different (see Supplementary Figure S4), except for fC3 fitting algorithm (*p* < 0.01). T2 differences were significantly different for all conditions. We cannot rule out a potential effect of the anesthesia conditions, since the gas mixture was slightly different between the two centers (2% in air at C2 vs. 2% in a mixture of Air and O_2_ (7:3). However, no T2 differences were found in an additional experiment in which the effect of breathing air or 100% O_2_ as carrier gas was tested. Another potential source of T2 difference between C1 and C2 may be that despite standard spoiling settings in the sequence, the T2 measurements may be affected by the presence of residual stimulated echo signal. Given the different transmit coils (single-channel for C1 and quadrature for C2), these signals may differ between C1 and C2 and account for differences found in T2 values.

Finally, we note that, in literature, T2 values generally seem more variable between centers than T1 values (see Figure 4). After examining multicenter data quality, we searched for possible differences when processing data with software developed in different centers.

### Image Processing Pipeline Comparison

The goal of this comparison was to explore whether data processing could be distributed to different centers using the solution locally developed rather than benchmarking them for selecting the best one. For relaxometry mapping, two main steps are required: fitting the raw data to extract relaxation times and segmenting the brain to compute mean values per brain area. Fitting data is clearly a simple mathematical operation. No consensus currently exists for this operation, and, as indicated in Table 1, differences exist in available solutions hosted in our three centers. Advantageously, the differences in fitting equation and in optimization methods did not impact the final results, neither for T1 nor for T2. Note that differences generated by the use of different fitting parameters were lower than those between the data acquisition centers and even those between data acquired at the same center.

For brain segmentation, we adopted a multi-atlas procedure. Multi-atlas techniques outperform single atlas approaches in accounting for individual structural variability (Wang et al., 2014). Here, we considered two implementations on two different platforms, BrainVISA and VIP, of the multi-atlas approach proposed in Lancelot et al. (2014) for rats. As mentioned in Table 1, some preprocessing steps differ: the registration [ANTS (Avants et al., 2010) vs. block-matching combined to Free Form Deformation (Lebenberg et al., 2010)] and the number of atlases used (11 vs. 12). Comparison of the parcelations obtained using the two methods revealed no differences in terms of parcel volume and DICE score. DICE were higher than 0.8 but for ventricles (see Supplementary Figure S5), suggesting and excellent overlap between parcelations. Differences were not significant when comparing T1 and T2 values obtained with the two multi-atlas segmentation procedures.

### Reference Maps

Publicly available quantitative reference maps for longitudinal (T1) and transverse (T2) relaxation times are valuable for investigating and monitoring deviations from normality in animal models of pathologies. Both T1 and T2 values have been reported in literature based on a limited number of animals. We extended these works by using a larger number of animals (*n* = 40) and by providing values in additional regions. Figure 4 shows the T1 and T2 values obtained in this study together with literature values. There is a good agreement between all T1 values. This is consistent with the low dispersion found between the two data providers (2%). The dispersion of literature values is larger for T2, especially when older studies are included. Similarly, we found a larger 9.5% dispersion between T2 values from our two data providers. Altogether, the values reported in this study are in good agreement with several recent publications (Del Bigio et al., 2011; Gigliucci et al., 2014; Suleymanova et al., 2014; Koundal et al., 2015; Liachenko and Ramu, 2017).

### Limitations

Data came from only two providers running similar MR systems from the same manufacturer. This is, however, very common in the preclinical imaging community. Data were acquired in only one animal strain (Sprague Dawley) and at one age (young adult). These two factors may indeed modify relaxation times (Knight et al., 2016; Liu et al., 2018). Additional changes in values may also occur in case of physiological changes (perfusion, oxygenation, temperature). In addition, our reference maps could be refined by including even more individuals and data could be reanalyzed to further limit the impact of stimulated echoes in T2 estimates, a potential source of bias. However, data acquired and processed with the same methods as those used in the study would remain comparable to the reference maps available from this study.

## Data Availability Statement

Raw data and reference T1 and T2 relaxometry maps, as well as processing pipelines are freely available on request from the corresponding author MD. Please refer to the present paper in case of the reuse of these datasets and pipelines.

## Ethics Statement

The animal study was reviewed and approved by the French Ministry of Agriculture (nos. 380945 and A3851610008 for experimental and animal care facilities for C1 and G130555 for C2).

## Author Contributions

TrD, FK, ThD, VN, EB, and MD contributed to the conception and design of the study and performed the data analysis. TrD, FK, EB, and MD acquired the data. TrD organized the database and pipelines. TrD and MD wrote the first draft of the manuscript. All authors contributed to the manuscript revision, read, and approved the submitted version.

## Conflict of Interest

The authors declare that the research was conducted in the absence of any commercial or financial relationships that could be construed as a potential conflict of interest.

## Footnotes

1 https://shanoir-ng-dev.irisa.fr/

2 https://www.creatis.insa-lyon.fr/vip/

3 https://www.ncbi.nlm.nih.gov/pmc/articles/PMC3085577/

4 http://www.fil.ion.ucl.ac.uk/spm

5 http://brainvisa.info/

6 Real Statistics Resource Pack software (Release 5.4). Copyright (2013 – 2018) Charles Zaiontz. www.real-statistics.com
