# Supplementary material for: A Multicenter Preclinical MRI Study: Definition of Rat Brain Relaxometry Reference Maps
Source: Front Neuroinform. 2020 May 19;14:22. doi: 10.3389/fninf.2020.00022 (PMC7248563; doi:10.3389/fninf.2020.00022)
Supplement: Supplementary file 1 [file Presentation_1.pdf]

Supplementary Material

Table 1: Animal information

Acq month	center	Rat_id	Weight (g)	Age (weeks)	Breath rate (bpm)	Temperature (°C)
**December**	GIN	S1	274	7		
**December**	GIN	S2	258	7		
**December**	GIN	S3	286	7		
**December**	GIN	S4	264	7		
**December**	GIN	S5	300	7		
**December**	GIN	S6	294	7	70-82	
**December**	GIN	S7	304	7	75-97	
**December**	GIN	S8	304	7	82	
**December**	GIN	S9	288	7	70-80	
**December**	GIN	S10	296	7	75-97	
**December**	CRMBM	S11	249,5	7	60	
**December**	CRMBM	S12	263,6	7	80-88	36,8
**December**	CRMBM	S13	267	7	30	34
**December**	CRMBM	S14	269	7	75	37,2
**December**	CRMBM	S15	273	7	65-75	37
**December**	CRMBM	S16	279,9	7	90	37,5
**December**	CRMBM	S17	277	7	80	37,7
**December**	CRMBM	S18	277	7	75-80	35,6
**December**	CRMBM	S19	271,3	7	65	34,6
**December**	CRMBM	S20	274	7	70	35,9
**May**	GIN	S21	294	10	70	37,7
**May**	GIN	S22	256	10	70-75	37,8
**May**	GIN	S23	278	10	70	38,5
**May**	GIN	S24	282	10	70	37,9
**May**	GIN	S25	268	10	65	36,8
**May**	GIN	S26	274	10	70	37,7
**May**	GIN	S27	314	10	70	37,5
**May**	GIN	S28		10	75	37,1
**May**	GIN	S29	262	10	60	36,8
**May**	GIN	S30	274	10	65	37
**May**	CRMBM	S31	297,5	7	60	34,8
**May**	CRMBM	S32	284,9	7	60	34,8
**May**	CRMBM	S33	302,7	7	55-55	35,3
**May**	CRMBM	S34	284,8	7	55-60	35,1
**May**	CRMBM	S35	290,5	7	70-60	35
**May**	CRMBM	S36	284,3	7	65	34,4
**May**	CRMBM	S37	275,7	7	60	34,5
**May**	CRMBM	S38	286,9	7	120-60	34,8
**May**	CRMBM	S39	285,5	7	120-60	36,2
**May**	CRMBM	S40	290	7	65	34,3
**Mean**			279.40 (SD=13.87)		67.13 (SD=11.42)	36.21 (SD=1.36)

Data

Inter subject variability

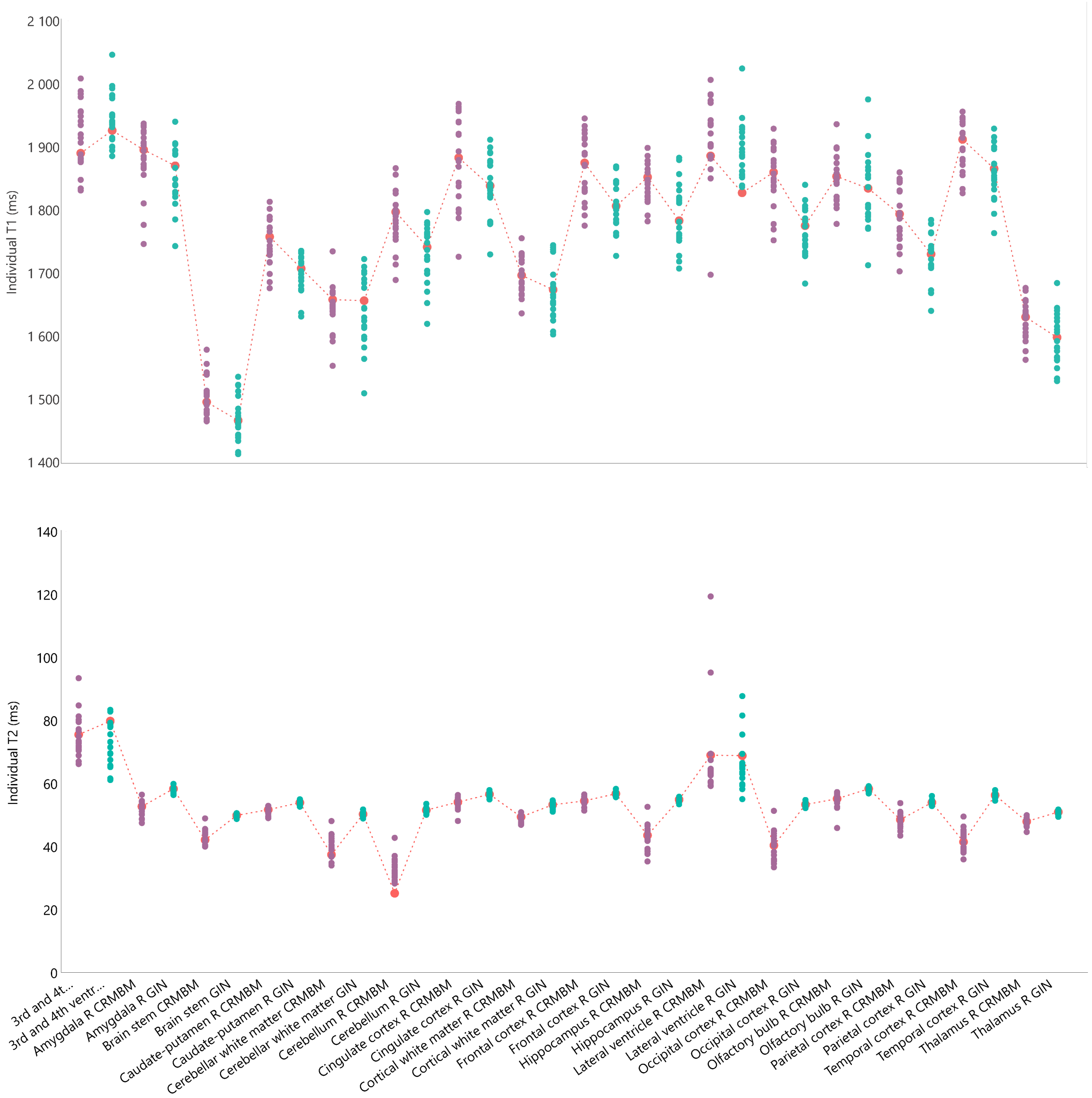

Figure S1: Individual relaxation time values for 13 regions of interest for the left hemisphere. Top: Individual T1 values. **Bottom**: Individual T2 values. Green circles for aC1 values; purple circles for aC2 values and corresponding mean values indicated with a red mark and dash-line. fC2 fitting pipeline and sC3 multi-atlas segmentation were used.

Inter-center variability

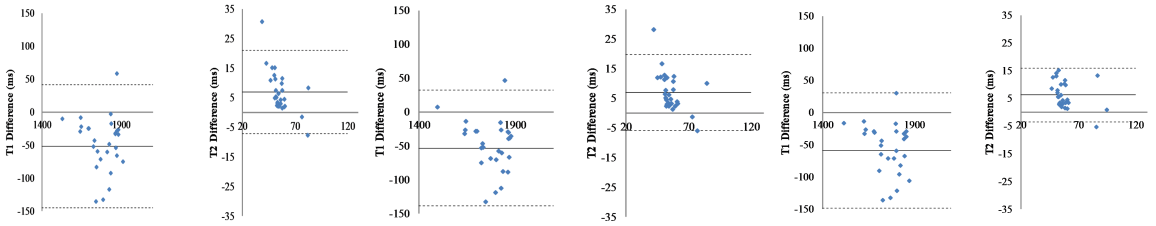

Figure S2: **Inter-center variability**. Differences between T1 (left) and T2 (right) relaxation times computed from data acquired at aC1 (n=20) and aC2 (n=20) for the 29 regions of interest. Fitting pipeline fC1 and Segmentation sC4 (left). Fitting pipeline fC2 and Segmentation sC3 (middle) Fitting pipeline fC3 and Segmentation sC4 (right). Solid line: mean difference. Dash lines: ± 2 standard deviation. X-axis in ms.

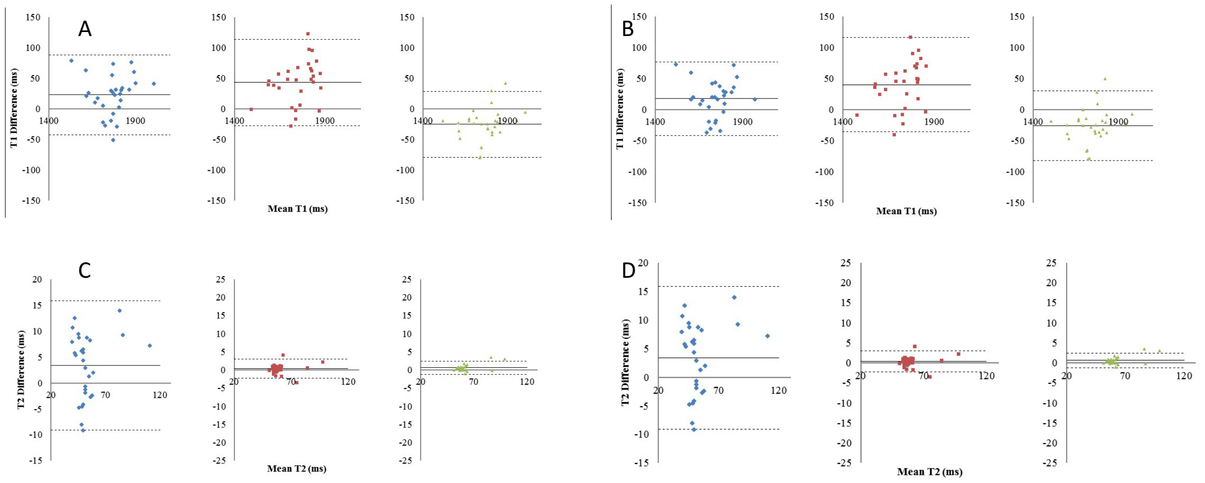

Figure S3. Intra-center reproducibility. Top T1 acquisition: Bland-Altman graph showing the differences in T1 relaxation time (mean values for each ROI) between the two acquisitions versus the corresponding mean T1 value using two processing pipelines. A) Left: S32 (aC2). Middle: S21 (aC1). Right: S22 (aC1). Fitting pipeline fC1, Segmentation sC4. B) Left: S32 (C2). Middle S21 (C1). Right S22 (C1).. Fitting pipeline fC3, Segmentation sC3.

Bottom T2 acquisition: Bland-Altman graph showing the differences in T2 relaxation time (mean values for each ROI) between the two acquisitions versus the corresponding mean T2 value. C) Left: S32 (aC2). Middle: S21 (aC1). Right: S22 (aC1). Fitting pipeline fC1, Segmentation sC4. D) Left: S32 (C2). Middle S21 (C1). Right S22 (C1). Fitting pipeline fC3, Segmentation sC3.

Solid line: mean value, dash lines ± 2 standard deviations.

Pipelines comparison

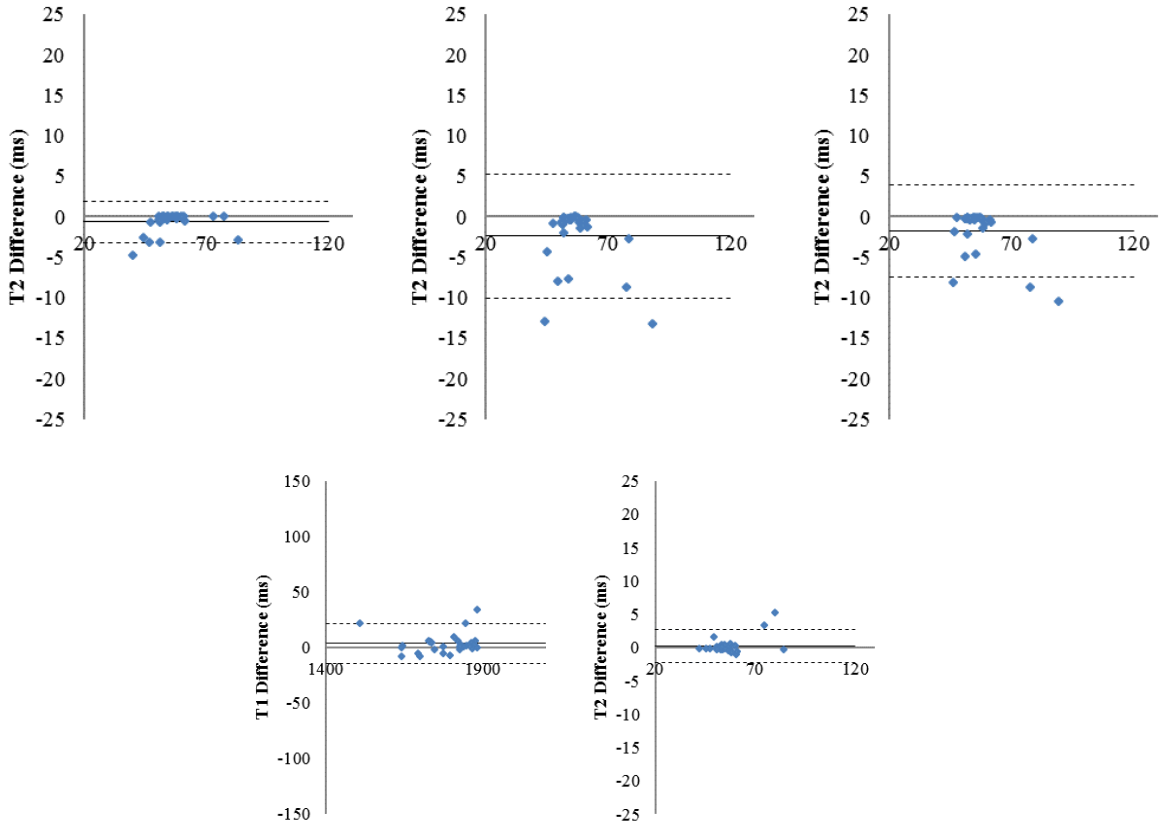

Figure S4: Comparison pipelines. Top: Fitting pipelines. T2 relaxation times values differences computed using different fitting pipelines for all regions of interest (n=29) and average out over the whole set of animals (n=40). Left: T2 values differences for fC1 minus fC2; Middle: T2 values differences for fC1 minus fC3; Right: T2 values differences for fC2 minus fC3. sC4 for segmentation. Solid line: Mean difference. Dash lines: ± 2 standard deviations. **Bottom: Segmentation pipelines.** T1 (left) and T2 (right) relaxation times value differences measured for all regions of interest and average out of the whole set of animals (n=40) using the two different segmentation pipelines sC3 and sC4. fC2 fitting pipeline. Solid line: mean difference. Dash line: ± 2 standard deviations.

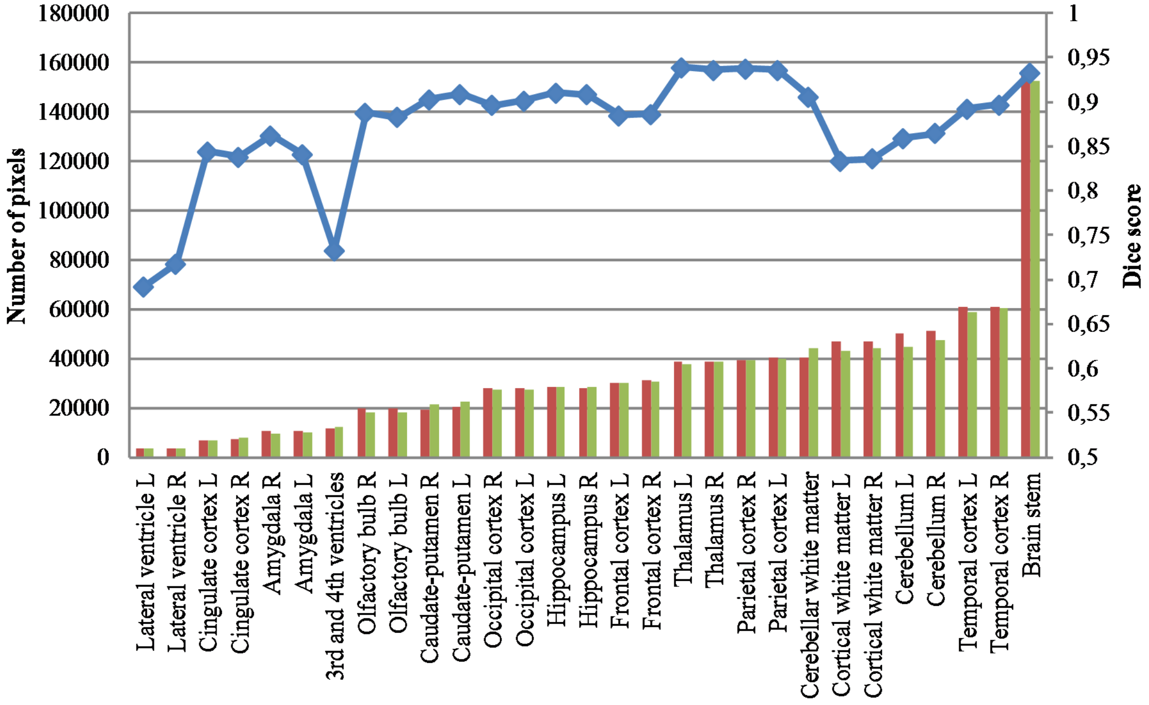

Figure S5: Differences between sC3 (Mircen) and sC4 (ICube) multi-atlas segmentation. Dice score (blue) and number of voxels using sC3 (green) or sC4 (red) in the different regions.
